# Using discrete choice experiments to measure preferences for hard to observe choice attributes to inform health policy decisions

**DOI:** 10.1186/s13561-020-00276-x

**Published:** 2020-06-11

**Authors:** Eline van den Broek-Altenburg, Adam Atherly

**Affiliations:** The Larner College of Medicine, University of Vermont, 89 Beaumont Ave, Burlington, VT 05405 USA

**Keywords:** Stated preferences, Choice modelling, Unobserved characteristics, Choice attributes, Discrete choice experiment

## Abstract

**Background:**

Models of preferences in health services research (HSR) and Health Economics are often defined by readily available information, such as that captured in claims data and electronic health records. Yet many important questions about patient choices cannot be easily studied because of a lack of critical data elements. The objective of this review is to outline the advantages of using stated preferences (SP) data in health services research, and to outline how these methods can be used to evaluate choices that have not yet been offered or studied.

**Main body:**

This article focuses on the application of DCE’s to relevant policy and health system delivery questions currently relevant, particularly in the United States. DCE’s may be helpful to collect data from patient or consumer data that we currently do not have. The article provides examples of research questions that have been answered using SP data collected with a DCE. It outlines how to construct a DCE and how to analyze the data. It also discusses the methodological challenges and emphasizes important considerations regarding the design and estimation methods.

SP data can be adopted in situations where we would like to have consumer choice data, but we currently do not. These are often hypothetical situations to analyze the decision-making process of individuals. With SP data it is possible to analyze trade-offs patients make when choosing between treatment options where these hard to measure attributes are important.

**Conclusion:**

This paper emphasizes that a carefully designed DCE and appropriate estimation methods can open up a new world of data regarding trade-offs patients and providers in healthcare are willing to make. It updates previous “how to” guide for DCE’s for health services researchers and health economists who are not familiar with these methods or have been unwilling to use them and updates previous description of these methods with timely examples.

## Background

Models of patient preferences in health services research (HSR) are defined by readily available information, such as that captured in claims data and electronic health records. Yet many important questions about patient choices cannot be easily studied because of a lack of critical data elements. In these questions, there are attributes of the choice that are important to patients but are not readily observed by researchers. For example, studies of hospital choice may lack data on measures of quality that matter to subgroups of patients more than crude measures like mortality such as the amount of face-to-face contact with health professionals, or logistical considerations such as parking.

One option to measure hard to measure choice attributes is to use stated preferences (SP) data. With SP data it is possible to analyze trade-offs patients make when choosing between treatment or policy options where these hard to measure attributes are important. Henscher and Bradly (1993) described stated preferences as “potential choices in terms of a set of constructed measures of attributes of real or hypothetical alternatives” whereas revealed preferences (RP) data are the “actual choices in terms of a set of market-based measurements of attributes of alternatives (which by definition are restricted to the currently available feasible set)” [1].

This paper outlines the advantages of using SP data in health services research to inform policy making and describes how and when to use a Discrete Choice Experiment (DCE) to collect SP data. It also gives examples of questions that have previously been answered using SP data in health economics and adds examples of potential areas of health services research that would benefit from using DCE’s, particularly in the United States.

SP data are commonly used in academic disciplines outside of healthcare to measure choice attributes or choices that are not easily observed. For instance, in transportation economics, stated preferences have been used to assess an individual’s willingness to make trade-offs in terms of time-savings, safety or frequency and reliability of the transport service and price [2–4]. Similarly, environmental economists have used stated preferences to analyze environmental valuation, such as tourists’ preferences for eco-efficient destination planning options [5, 6]. Researchers in health services research analyze similar choice attributes related to accessibility and costs, but, other than those who use it for cost-effectiveness analysis, do not yet widely embrace SP data as much as other fields.

### How SP data is being used: economic evaluations

The collection and use of SP data in health is much more common in European countries and Australia than in the United States [7]. SP data is most frequently used to develop more accurate disease-specific utility-based health outcome measures. Preference-based quality of life instruments such as the EQ-5D, ICECAP-O and the ASCOT are valid and reliable measures, but often not the most appropriate instruments in some disease-specific or individual-specific cost effectiveness studies. Thus, a common option is to collect SP data from patients to augment more generic health status instruments to get more appropriate estimates of quality of life. For example, Nieboer et al. discuss the OPUS and ICECAP measures, which are quality of life indexes for elderly persons, and tested the measures in a DCE among the elderly. They concluded that these measures did not include relative values of different services for different patients [8]. Similarly, a study modified existing profile measures relevant to glaucoma and developed a six-dimensional profile instrument that was used to create a glaucoma-specific preference-based utility measure using SP data [9].

### How SP data could be used

In health services research, the quality of SP data is often evaluated relative to RP data, which is the gold standard. RP data models assume that the preferences of consumers are revealed by their purchasing habits. RP data is viewed as having higher reliability and face validity, as it portrays the world as it is (e.g. the current market equilibrium). RP data can be used to analyze choices made by individuals and compare the effect of policy changes on consumer behavior [10].

SP data can be adopted in situations where we would like to have consumer choice data, but we currently do not. These are often hypothetical situations to analyze the decision-making process of individuals. For example, Fung and colleagues (2019), recently used a SP design to estimate that insurance premiums would increase by 4–7% if tax penalty for noncompliance with the Affordable Care Act’s individual mandate were eliminated [11]. In this hypothetical context, RP data were not available; the study showed what would happen if such a policy were enacted.

SP data are also often used to analyze taste heterogeneity for different health services or products. In the policy debate about whether flavors should be banned in cigarettes and e-cigarettes, a study analyzed policy-relevant estimates of impacts of alternative flavor bans on preferences and demand for cigarettes and e-cigarettes in adult smokers and recent quitters [12]. A related study using SP data found that adult smokers’ demand for e-cigarettes is motivated more by health concerns than by the desire to avoid smoking bans or higher prices [13].

SP data can also be used when choices depend on the information context, such as a recent insurance choice study by McGary et al. [14]. In this study, the authors used an experiment with hypothetical Medicare Part D plan choices to test the effect of simplifying the default amount of financial information provided on the Medicare Plan Finder on respondents’ ability to select low-cost plans. They found that reducing the amount of financial information provided led to the selection of lower-cost plans, with no accompanying decrease in average plan quality or pharmacy network size, and an increase in the take-up of convenience options such as a mail-order pharmacy. The study showed that real-life choices like using interactive plan menus produce different results in different choice environments: when simplifying the cost attribute in an experimental setting, respondents chose more optimal cost-benefit plan options from an objective or rational choice perspective.

SP data can thus be used to get a better understanding of the trade-offs patients or policy makers make in the decision-making process. In the United States, SP data can contribute by measuring patient preferences for hard to observe choice attributes. This is particularly relevant because of efforts to make fundamental changes to the healthcare system, moving from volume-based to value-based healthcare delivery. Existing datasets can help analyze changes in spending and outcomes following these recent reforms but lack data on patients’ willingness to accept value-based care. SP data can fill that gap and address questions related to “patient centered” care, improved communication and the patient-provider relationship.

It is in this context that SP data may be useful to predict acceptance of changes based on attributes of a health product or service. Defining value from the patient perspective is particularly important in health systems where reform is focused on shared decision-making between physicians and patients. In the context of value-based care, SP data can also help analyze how decision-makers prioritize efforts to improve value. For example, when evaluating how community health teams set priorities and how they make trade-offs between considerations related to health, health equity or financial resources.

### Applying the right methodology- collecting SP data with a discrete choice experiment

An effective method to collect SP data is a Discrete Choice Experiment (DCE). A DCE provides the opportunity to estimate pair-wise choices and analyze marginal values or the total value of a health service or good. For example, a study examining the effect of reducing waiting times in the provision of rheumatology services used a SP elicitation technique to estimate the monetary value the waiting time reduction (as well as other possible changes) [15]. The unique contribution of a DCE is that it allows researchers to analyze the trade-offs that patients are willing to make including options that may not exist but could in the future*.*

Even though using DCE’s to elicit preferences for health outcomes is a great advancement from one-size-fits all instruments such as the EQ-5D, ICECAP-O and the ASCOT, those applying these methods still base their estimation on the underlying assumptions of the random utility model. This leads to limitations such as:

1. the trade-offs are often defined by the willingness-to-pay (WTP) measure alone, which is just one piece of an equation that represents the trade-offs people, and;
2. methods do not always account for individual preference heterogeneity depending on the estimation methods used, and;
3. the measurement methods, used in health economic evaluation, often insist on linear, additive utility.

This updated “how to” guide describes in more detail how the various design choices relate to the question of interest and how methods may and should differ when applying this framework to other, non HTA-related, healthcare questions.

## Methods

A DCE is a survey instrument that clearly explains both a baseline or status quo situation and alternatives that can be chosen. The survey starts with a description of a health product or service to be valued, the cost of the service (e.g. a premium for health insurance), the value elicitation questions or discrete choice sets based on attributes and levels, potential follow-up questions (e.g. to check responses or to get information about the way respondents processed the attributes) and auxiliary questions (e.g. demographics or behavioral questions). A DCE assumes healthcare services can be described by their attributes and that an individual’s valuation of choices depends upon the levels of these attributes. This way, instead of asking a patient “would you prefer treatment A or treatment B”, a DCE asks “imagine you have the choice between treatment A and B. A and B differ in the following ways ( ….) Would you choose A or B?” This allows the researcher to tease out the relative utilities of the attributes of the treatment.

Alternatives may be labeled (Bus, Car, Train) or unlabeled (Drug A, Drug B and Drug C), and the number of alternatives usually varies between two and six, depending on the choice scenario. In some cases, a “no choice” or “status quo” alternative may be offered. This may be relevant in situations when researchers are asking choice questions about a drug or a treatment that is not yet available. In the example that follows, patients are asked to choose between different locations for diagnostic services where they will compare two options that do not yet exist to their usual “status quo” source of care. The aim of this paper is to explain how these methods can be used to inform policy making, by using a practical example of a major change in a hypothetical health care delivery system.

### Example: optimal health system capacity design

Health systems, especially in the value-based context, are increasingly rewarded for keeping people healthy and out of the hospital, rather than for the volume of services provided. One focus in redesigning care delivery in the value-based context has been on decentralizing services, including higher-acuity care, into the outpatient environment. In this section, we will work through a DCE to inform decision-making regarding the allocation of resources or the restructuring of care delivery systems. In this example, a health system’s goal is to decentralize diagnostic radiology services by offering additional services away from the tertiary care center. The aim is to deliver diagnostic services in a way that is more convenient for the patient and lower cost for the system, while offering the same level of quality. The health system decided to set up a pilot project to test diagnostic services options, where alternative A is a mobile option for diagnostic services, alternative B is a fixed kiosk for diagnostic services and option C is the status quo of services in the medical center. The decision makers want to find out which attributes of diagnostic services patients care about the most. Existing data from electronic health records and claims data cannot predict choices for a product that does not yet exist, which suggests the use of a DCE. The next sections will walk through the steps of setting up a DCE in this example and will briefly explain some estimation methods.

### Attributes and levels - how to choose

Setting up a DCE, the first step is to describe the choice scenario. It is important to explain the “status quo” and the alternative choices. This involves defining the different attributes (what parts of the choices differ) and the “levels” of the attributes (what actual values for each attribute are presented to the respondent). Defining the attributes and levels is typically based on a combination of theory and prior literature. This initial piece of a DCE design may seem the most straightforward, but attribute generation for DCEs is often poorly reported, and it is unclear whether this element of research is conducted rigorously. Increasingly, researchers are careful to get the attributes and levels “right” [16–19].

Various methods have been used to define attributes for DCE’s, including literature reviews, theory or existing health outcome measures. Other methods also include patient surveys, text mining, and machine learning to “scrape” patients’ online platforms such as Reddit, or a combination of these methods. In addition, many studies start by conducting focus groups of patients who are similar to the planned DCE sample. For example, the study described earlier focused on estimating a utility-based glaucoma health outcome measure first conducted a focus group of glaucoma patients to identify important ways that glaucoma affected quality of life. The focus group results then were used to modify the existing profile measures relevant to glaucoma and develop a six-dimensional profile instrument. The qualitative, exploratory work is important to guide subsequent phases of the study; the advantages and disadvantages of different qualitative methods for developing attributes in health has been described at length by Coast and colleagues [17, 20].

Just as important is the choice of levels. The levels may also draw on any of the methods mentioned above, including qualitative data. In a DCE, the attribute levels are used to operationalize the alternatives included in the choice sets. It is important for the statistical analysis and interpretation of the data and results to select attributes and attribute-levels that properly describe the health care product or service. If the levels are not defined in the appropriate range, the estimated coefficients could be biased.

In our example, focus groups across the state, both in urban and rural areas, can be used to provide information about the key attributes and levels that are relevant to diagnostics services. This qualitative work can be supported by information from electronic health records of patients in the medical center, as well as a review of the literature regarding preferences for diagnostic services. Table 1 shows the attributes and levels that we defined for this example. Note that these are just a selection of attributes that could be tested; other factors such as a parking options at facilities may also matter. The more attributes and levels, the more complicated the DCE design will become. We will explain this in more detail in the next section.

**Table 1:** Attributes and levels in diagnostic services-study

Attributes	Levels
Hours of operation	9 am-5 pm; 6 am-9 pm; 24 h
Wait time to be seen	Same day; 3 days; 1 week
Wait time for results	24 h, 48 h; 1 week
Online scheduling	yes / no
Distance to center	10 M; 50 M; 100 M; 400 M
Co-pay / Out-of-Pocket costs	$0; $50; $150; $500

### Constructing feasible sets of attribute-level combinations

There are constraints in constructing feasible sets of attribute-level combinations. In our example, we could ask respondents to choose between alternatives where alternative A would have: 24 h-service; patients would be seen same day; wait time for the results is less than 24 h; copay equals zero; and they would have an online scheduling option. Unless respondents have compelling reasons for not choosing a mobile service, this alternative would be dominant. The goal of a DCE is to understand what trade-offs respondents are willing to make. A choice set like this does not require tradeoffs and therefore will not provide relevant information. This is referred to as a “dominant alternative” [21]. In this situation, the marginal effect of different attributes cannot be empirically estimated. Dominant alternatives are therefore excluded from choice sets.

Similarly, constraints related to realistic choices are important to consider. For example, it is unlikely that diagnostics services would be offered every day, 24 h a day, next to everyone’s house with zero out-of-pocket costs. Therefore, it is important to establish a feasible set of attribute combinations and exclude the potentially implausible combinations. Once feasible sets of plausible combinations are chosen, the analysis can be focused on trade-offs. “Unacceptable levels” are described in detail by Green and colleagues (1988) and refer to levels which are sufficiently high or low that the respondent will ignore other attributes and discard that option [22].

### Efficient design

Once the number of choice tasks has been selected and the dominant options, repeats and implausible attribute combinations excluded, researchers need to select which choices to present to survey respondents out of the multitude of possible choices. It is impossible to elicit consumer’s preferences for all possible combinations of the levels of the attributes, such as in a full factorial design, when a good or service has a multitude of identified attributes and levels. For example, consider a choice task with five attributes. If four of these attributes had three levels, while the fifth had two (yes or no), this would give rise to 162 possible scenarios (3*3*3*3*2) in a full factorial design. These 162 scenarios can then be combined into 1,291,040 potential choice set combinations with three alternatives in each choice set. In practice, it is impossible to have respondents rank over 1 million possible combinations of the attributes.

There are three ways to reduce the dimensions of the full factorial design matrix to fractional factorial designs: random designs, orthogonal designs, and efficient designs. With a random design, choice tasks are randomly assigned to respondents for each of the experimental conditions of the full factorial design. Random designs require more respondents than some other designs and may introduce higher standard errors because some choice sets will provide better information than others.

The alternative design approaches allow the user to specify combinations that should not appear in the design [22–24]. These are called efficient designs and can be created without dominant or repeating choice tasks. A design is considered more efficient if it can produce more efficient data in the sense that more reliable parameter estimates can be achieved with an equal or lower sample size. Where orthogonal designs minimize correlations between the attributes to zero; D-efficient designs are aimed at minimizing (co) variances of parameter estimates; A-efficient designs only looks at variances not covariances; and S-efficient is a sample size efficient design aiming to minimize the sample size needed to obtain statistically significant parameter estimates. These measures are “prior” estimates which can be based on researchers’ prior expectations on what the estimated model parameters will be based on literature or sample data. The researcher specifies utility functions that include these “priors”, and these are used to determine the logit probabilities and the log likelihood functions. In our example, the utility function for diagnostic services, where expected overall utility U of respondent *i* from diagnostic center *j* at the *c*^th^ choice situation is given by:

1 $$ {U}_{ijc}={\beta}_{1i}{Hours}_{ijc}+{\beta}_{2i} Wait\kern0.6em {Time}_{ijc}+{\beta}_{3i} Online\kern0.6em {Scheduling}_{ijc}+{\beta}_{4i}{Distance}_{ijc}+{\beta}_5{OOP}_{ijc}+{\varepsilon}_{ijc} $$

The level of efficiency then uses the prior parameters to select the optimal choice set. For example, the prior parameter for wait time could be − 0.018 and the prior for online scheduling 0.50. The S-efficient design focuses on sample size and could, for example, indicate that 750 respondents are needed if there are 12 choice tasks with 4 attributes and 3 levels per attribute in a design.

Once the priors are established and the utility functions are defined, there is software to help create the efficient design, such as NGene, which is proprietary, and AlgDesign, which is open source R code. Ngene allows the inclusion of the specific priors in the utility functions. The advantage of the Ngene algorithm is that it searches for a list of choice sets in which dominant alternatives do not appear, choice sets are not repeated, and the number of choice sets for which the answer can be inferred from the previous one is minimized. NGene reports the A-,S- and D-error per design options, which are model-specific. So, a 0.2 may be very good in one model, but poor in another. Overall, the lower the error, the better, as it is a measure of efficiency of the design and the amount of “noise”. In our example, priors for the parameter estimates can based on the literature and clinical expertise and an efficient design can be created. An example of one of the choice sets is shown in Table 2.

**Table 2:** Example of One Choice Alternative in DCE

	Alternative 1	Alternative 2	Alternative 3
**Hours of Operation**	9–5	6–9	24 h
**Wait time to be seen**	3 days	1 day	1 week
**Distance to center**	10 M	150 M	50 M
**Online scheduling**	No	No	Yes
**Co-pay / OOP**	$50	$0	$500

One other important design attribute is the number of choice tasks. In some cases, a study may contain more choice tasks than the researcher wants to ask respondents. In this case, a blocking experimental design can be used [25, 26]. Blocks are subsets of the choice questions, which are usually equally sized, that contain a limited number of choice questions for each respondent. In those cases, respondents are randomly assigned to a block and answer the choice questions in that block instead of the entire design.

### Defining the sample and collecting the data

After defining the choice sets, including the attributes and levels, the next step is to define the sample. Ensuring that a DCE has a sufficient sample size for statistical power is important, but this generally requires smaller samples than many studies in health service research. The different software programs described previously can define the optimal sample.

There are several methods to collect the data. Collecting large numbers of respondents is often timely and/or costly and involves a trade-off between sample size and the cost of the survey. Some researchers in the social sciences use Amazon’s Mechanical Turk (MTurk) or similar online platforms to collect survey participants. MTurk is an online labor market created by Amazon to assist “requesters” in hiring and paying “workers” for the completion of computerized tasks, most often surveys. Increasingly, empirical publications are based on data using MTurk [27–30].

Another data collection route is to use a company specialized in surveying such as SAP’s Qualtrics, who also provides the software to design the survey. Respondents are recruited by the organization through website intercept recruitment, member referrals, targeted email lists, permission-based networks, and social media. Census statistics for variables such as gender, age, race or living area may define the sampling strategy.

### Estimation methods

Econometric modelling of SP data in a DCE usually involves simplifying decision heuristics and relies on a number of assumptions, such as random utility maximization. Many discrete choice frameworks are based on random utility theory where an individual’s utility for a choice alternative is assumed to consist of a deterministic component and a random utility component. Given the principle of utility maximizing behavior, the probability of choosing a choice alternative is then equal to the probability that its utility exceeds that of all other choice alternatives in the choice set.

There are several well-established methods for analyzing discrete choice data. If the research question relates to whether preferences vary among people with different individual characteristics, a multinomial logit model can be used. Conditional logit models are appropriate when variables vary over the alternatives. Conditional logit relates the probability of choice among two or more alternatives to the characteristics of the attribute levels defining those alternatives [31]. However, these models exhibit the strong assumptions of independent and identically distributed error terms and the independence of irrelevant alternatives (IIA). In a DCE, the elements describing the alternatives are the attribute levels used to define each profile in the choice task. Thus, IIA is addressed in the DCE design.

In a nested logit model, the choices are grouped into nests (clusters) where IIA holds within a nest but not necessarily between nests [32]. A mixed logit model accommodates even more flexible substitution patterns, and allows for random taste variation, unrestricted substitution patterns, and correlation in unobserved factors [33, 34]. Mixed logit models make it feasible to derive individual-specific estimates conditional on the observed individual choices [35]. The model assumes a specific distribution for each random (taste) coefficient and distributions can vary across the coefficients.

Modelling data from a DCE may also involve including interactions of choice attributes and consumer characteristics. These interactions can be used to test to what extent differences in preferences can be explained by differences in the observed characteristics of the respondents. Whether to include or not to include interaction terms generally requires either theory, intuition, and feasibility in terms of sample size and survey-design parameters. For example, some of the systematic differences between respondents’ preferences for diagnostics services may be explained by their age. This can be used to test sub-hypotheses like “Older people are more likely prefer a shorter distance to the service”, or “Higher educated prefer an online scheduling tool”.

## Conclusions

Many studies of patient choices in healthcare are hampered by a lack of critical data elements. For some questions, there are attributes of the choice that are important to patients but are not readily observed by researchers. One approach to this lack of data regarding attitudes, preferences and choices is to use SP data. An effective method to collect SP data and study patients’ trade-offs is a DCE. This article focused on the application of this technique to relevant policy and health system delivery questions currently relevant, particularly in the United States. The use of SP data may not be helpful in many situations, but in the U.S., they are barely used at all. DCE’s may be helpful to collect data from patient or consumer data that we currently do not have.

In this article, we gave examples of research questions that have been answered using SP data collected with a DCE. Most DCE’s today are being conducted in Europe and many of those studies are being used for cost effectiveness (CEA) and cost-benefit analysis. This article emphasized that DCE’s can be used for many other applications in health, beyond improving utility in CEAs. The amount of DCE papers used for the systematic review in the Unites States is small compared to the use in European and Australia. This is even more evident in the broader context of applications in transport and other subfields of economics.

We explained that the use of SP data and DCE’s to study preferences in healthcare comes with important methodological challenges. Especially the hypothetical character makes it challenging to use SP data for out of sample predictions. Nevertheless, a carefully designed DCE and appropriate estimation methods can open up a new world of data regarding trade-offs patients and providers in healthcare are willing to make.

SP methodologies have the potential to inform health policy yet are underutilized. This represents an important missed opportunity for the research community. In health research, the quality of SP data is often evaluated relative to RP data, which is the gold standard. Where RP data models assume that the preferences of consumers are revealed by their purchasing habits and can be used to analyze choices made by individuals and compare the effect of policy changes on consumer behavior; SP data can be adopted in situations where consumer choice data does not exist. These are often hypothetical situations to analyze the decision-making process of individuals for policy options that may not currently exist and analyze trade-offs patients make when choosing between treatment options where these hard to measure attributes are important. Our work contributes to the literature about alternative data sources for health services research and health economics.

The key new contribution of this paper is as a comprehensive “how to” guide for DCE’s in health for health services researchers and health economists in the U.S. who are not familiar with these methods. This paper also serves as a resource for those who are interested in using SP data to answer questions about patient preferences and choices which cannot be easily studied because of a lack of critical data elements in secondary data.

## Data Availability

There are no datasets generated or analyzed during the current study.

## Abbreviations

ASCOT: Adult Social Care Outcomes Toolkit
CEA: Cost-Effectiveness Analysis
DCE: Discrete Choice Experiment
EQ-5D-5 L: EuroQol 5-Dimensions Questionnaire with 5-Level scale
HSR: Health Services Research
ICECAP-O: ICEpop CAPability measure for Older People
IIA: Independence of Irrelevant Alternatives
MTurk: Amazon’s Mechanical Turk
OPUS: Older People’s Utility Scale
RP: Revealed Preferences
SP: Stated Preferences
